# Time to Deal with Rheumatoid Cachexia: Prevalence, Diagnostic Criteria, Treatment Effects and Evidence for Management

**DOI:** 10.31138/mjr.33.3.271

**Published:** 2022-09-30

**Authors:** Elisavet Efthymiou, Maria G. Grammatikopoulou, Konstantinos Gkiouras, Georgios Efthymiou, Efterpi Zafiriou, Dimitrios G. Goulis, Lazaros I. Sakkas, Dimitrios P. Bogdanos

**Affiliations:** 1 Department of Nutritional Sciences & Dietetics, Faculty of Health Sciences, International Hellenic University, Alexander Campus, Sindos, Thessaloniki, Greece,; 2 Department of Rheumatology and Clinical Immunology, Faculty of Medicine, University General Hospital of Larissa, School of Health Sciences, University of Thessaly, Larissa, Greece,; 3 Department of Dermatology, Faculty of Medicine, School of Health Sciences, University of Thessaly, Biopolis, Larissa, Greece,; 4 Unit of Reproductive Endocrinology, 1st Department of Obstetrics and Gynecology, Medical School, Faculty of Health Sciences, Aristotle University of Thessaloniki, Thessaloniki, Greece

**Keywords:** testosterone, hypogonadism, body cell mass, oral nutrient supplementation, methotrexate, disease activity, sarcopenia, DAS28, fat-free mass, body composition

## Abstract

Cachexia is an early result of rheumatoid arthritis (RA) (rheumatoid cachexia, RC), characterised mainly by involuntary loss of fat-free mass. RC is apparent in 1–67% of patients with RA, depending on the diagnostic criteria applied and the method used for the assessment of body composition. RC is associated with increased inflammation and disability, lower health perception, and greater mortality risk. These changes in body composition are driven by the inflammation process, the low levels of physical activity, the underlying testosterone deficiency and hypogonadism, and the pharmacotherapy required for RA. Chronic inflammation enhances resting energy expenditure as a response to stress, inducing an energy deficit, further propelling protein turnover. The use of corticosteroids and tumour necrosis factor α (TNF-α) inhibitors tend to increase fat accumulation, whereas other disease-modifying antirheumatic drugs (DMARDs) appear to induce increments in fat-free mass. The present review presents all information regarding the prevalence of RC, diagnostic criteria, and comorbidities, as well as the effects of pharmacotherapy and medical nutrition therapy on body composition of patients with RA.

## INTRODUCTION

Rheumatoid arthritis (RA) belongs to the most prevalent chronic inflammatory autoimmune diseases, affecting joints, but also several other body organs, including the heart.^1^ The global prevalence of RA has been estimated at approximately 0.69% of the total population,^2,3^ with distinct environmental differences, as a greater incidence is observed among inhabitants of urban areas.

Cachexia in RA (rheumatoid cachexia, RC), is mainly characterized by loss of muscle mass, in particular appendicular lean mass (ALM), and associated with accumulated fat mass (FM), situated mainly in the trunk area,^4–8^ indicating a shift towards the development of abdominal obesity.^9^ According to Rall and Roubenoff,^10^ loss of body cell mass (BCM) consists of an important issue of concern for patients with RA. BCM consists primarily of muscle and visceral mass (erythrocytes, serum proteins, lymphocytes, etc.), and is the part of the body with the greatest metabolic activity (95% of the total activity), determining protein requirements, energy expenditure, and the metabolic response to stress.^10,11^ In RA, patients lose between 13–15% of their BCM,^12,13^ an amount corresponding to 1/3 of the BCM volume that is associated with mortality.^10,14–16^ Thus, the increased mortality observed in patients with an RA diagnosis may be the product of altered body composition and subsequently, RC.

The prevalence of RC is high and it has been estimated that approximately 1/3 of the patients with an RA diagnosis develop cachexia.^17^ Body mass index (BMI) is not an appropriate tool for the detection of RC, as body fat (BF) may be within normal levels, or increased.^18,19^ Furthermore, different BMI cut-offs have been suggested for patients with RA, reduced by 2 kg/m^2^ for each weight status tier, to better depict the changes in body composition due to RC.^20^ RC appears to be the result of several synergistic mechanisms, including an excessive production of inflammatory cytokines and hypermetabolism.^21^ Hypermetabolism in particular is the result of increased protein degradation and decreased muscle mass,^13,21^ which, in the presence of low physical activity levels and sedentary lifestyle, result to an increased accumulation of FM,^22–24^ further propelling inflammation.^25^ Moreover, the underlying testosterone deficiency and hypogonadism, paired with the observed decrease in the production of insulin and the intake of GC medication, pave the way for the development of RC.^21,22,26–28^

The limited available studies on the effect of pharmacotherapy on RC suggest that the intake of corticosteroids (CG) and tumour necrosis factor α (TNF-α) inhibitors tend to increase FM accumulation, whereas the use of tocilizumab (TCZ) might induce a gain in lean body mass (LBM).^29,30^ These adverse changes in body composition are observed early on during the disease process.^5,6^

In rheumatology clinics, the assessment of body composition consists of a neglected issue,^5^ with most rheumatologists requiring further capacity building on the subject, or being too busy to screen patients. As a result, RC is often undiagnosed, unrecognized, and untreated.^5^ However, changes in body composition are not the only signs associated with RC, as underlying inflammation is actually propelling the development of RC, with pharmacotherapy acting in parallel and synergistically towards this goal, although the exact mechanisms have not yet been delineated. Patients with RC demonstrate increased pro-inflammatory cytokines, TNF-α and interleukin-1β (IL-1β) concentrations,^17^ further complicating the management of RA.

Lately, RC gained research interest with many studies being conducted, while providing evidence for its effective treatment. The aim of the present review was to synthetize all available scientific data to date regarding RC, its diagnosis, prevalence, associated factors and possible treatment modalities.

## EPIDEMIOLOGY: PRIMARY RESEARCH ON THE PREVALENCE OF RC

Several case-control studies evaluated muscle-mass loss in patients with RA against healthy controls, with many evaluating sarcopenia, or myopenia instead of cachexia. Giles and associates^31^ revealed that RA status is associated with greater odds of sarcopenia, excess in BF, and sarcopenic obesity in women, but not men. Distinct body composition phenotypes were more evident among patients in the normoweight BMI category. Furthermore, specific RA characteristics, such as increasing joint deformity, patient-reported disability scores, elevated c-reactive protein (CRP) concentrations, rheumatoid factor seropositivity, and a lack of current treatment with disease-modifying antirheumatic drugs (DMARDs) were associated with abnormal body composition phenotypes.^31^ Ecici^32^ noted that 31.5% of the newly diagnosed, treatment-naive patients with RA exhibited sarcopenia, with a greater prevalence among men. Furthermore, 3 months of DMARD treatment appeared to reduce this prevalence, although grip strength was further reduced since diagnosis.^32^

On the other hand, in a Chinese population, Lin et al. ^33^ evaluated the prevalence of myopenia in patients with RA against age- and sex-matched, healthy controls. Compared with the control arm, patients with RA with normal BMI exhibited a higher rate of myopenia and overfat, and a greater BF as a percent of body weight (BW). **Table 1** details the primary studies assessing the prevalence of RC among patients with RA. Most studies had a cross-sectional design,^12,18,34– 39^ one was a one-year prospective cohort^17^ and the remaining were case-control studies, with the control group comprising either of healthy participants^40–42^ or RC-free patients with an RA diagnosis.^22,43^ Among patients with an RA diagnosis, the overall prevalence of RC ranged from 1% in the Netherlands^35^ to 67%.^12^ In a recent meta-analysis, Santo17 calculated the pooled prevalence of RC at 19% (95% confidence intervals [CI]:07–33%), with a greater prevalence (29%) among studies using dual-energy x-ray absorptiometry (DXA) as the method for assessing body composition. However, in this meta-analysis, one study using the exact same sample was included twice (from two distinct publications),^44,45^ indicating a unit-of-analysis issue according to the Cochrane Collaboration.^46^ Moreover, several studies identified herein, were not included in the aforementioned meta-analysis, as the ones conducted by Hugo,^41^ Roubenoff,^12^ Santillán-Díaz,^22^ Müller,^40^ Pineda-Juárez,^39^ and the most recent ones published by Santo,^47^ Ångström^38^ and Papichev.^37^ The wide CI suggested by Santo and associates^17^ meta-analysis reflect the great heterogeneity of the pooled studies, the variety in disease progression observed in participants and the different methodology used to assess RC. Collectively, the available research indicates that studies of better quality are required to assess the prevalence of RC.

**Table 1.** Primary studies assessing RC in patients with RA.

**First author**	**Study**				**Participants**			**RA Diagnosticcriteria**	**Body composition method**	**RC**		**Results**
	**Origin**	**Design**	**Duration**	**Recruitmentsite**	**N (% women)**	**RR (%)**	**Age (years)**			**Diagnostic criteria**	**Prevalence (%)**	
Hugo^41^	France	CC	2010–4	Bordeaux University Hospital	57 (73%) patients with RA and 7 healthy controls	NR	57±10^MSD^	ACR	DXA	Engvall et al. ^18^ and Elkan et al*.* ^44^	18 (Engvall) and 28 (Elkan)	Low levels of PA and treatment by GC were associated with the nutritional complications of RA. Patients with RC had low FFM and high % fat. MS and RC were not associated with each other. Hypermetabolism is suspected as the cause of RC.
Lombard^34^	S. Africa	CS	NR	Tygerberg Hospital and Groote Schuur Hospital	251 (83%) patients with RA	NR	57.4±13.6^MSD^	NR	Skinfolds (Durnin and Womersley) and the Siri equations	Elkan et al. ^44^	12	Nearly half of the studied population with RA was classified as obese (45.9 %) and a further quarter as overweight (26.8 %). RC was present in some patients not classified as obese.
Van Bokhorst^35^	The Netherlands	CS	2011	Department of Rheumatology at the VU Medical Center	103 (79%) consecutive patients with RA	NR	26–90^R^	NR	BIA	Evans et al. ^48^	1	The recently proposed definitions of both ‘pre-cachexia’ and ‘cachexia’ are not clinically useful to identify and diagnose impaired nutritional status in RA patients treated with currently available anti-rheumatic drugs.
Santillán-Díaz^22^	Mexico	CC	NR	Rheumatology Clinic at the Instituto Nacional de Rehabilitación	94 (92.55%) patients with RA, 20 as cases (with RC) and 74 as RC-free patients	NR	51.4±13.4^MSD^	ACR/EULAR^116^	BIA (Systems Quantum X, Clinton Twp, Mi, USA)	a vector analysis method (BIVA Software 2002) NOD^117^	21.3	Association between RC and disease activity was not observed, the interaction of folic acid and methotrexate consumption behaved as a protective factor for the presence of RC.
Metsios^43^	UK	CC	NR	Department of Rheumatology of the Dudley Group of Hospitals	34 patients with RA and RC and 366 patients with RA (RC-free), all from the DRACCO cohort (73%)	NR	54–73^R^	NR	BIA (Tanita BC418MA, Tokyo, Japan)	Engvall et al. ^18^	8.5	FFM and albumin levels were decreased in the RC group, but BF (%BW) was not different compared to the RC-free patients. No differences were detected in either the classical, or novel CVD risk factors, 10-year CVD risk or the prevalence of established CVD.
Maghraoui^36^	Morocco	CS	June–December 2013	Military Hospital of Rabat	178 patients with RA (82.6%)	NR	54.1±11.5^MSD^	ACR	DXA (Lunar Prodigy)	Engvall et al. ^18^	53.9 (53.7% women, 54.8% men)	Between women with and without RC, those with RC had a longer disease duration, greater disease activity, higher steroid cumulative dose, and a greater prevalence of erosive arthritis. Half of the patients with RA may have RC with a normal/high BMI, this condition was associated with disease activity and low hip BMD, but not with VF.
Elkan^45^ and Elkan^44^	Sweden	CS	NR	Rheumatology Department, Karolinska University Hospital Huddinge	80 consecutive outpatients with RA (76%)	NR	61^M^	NR	DXA (GE-Lunar Prodigy) and BIA (Tanita Corp., Tokyo, Japan)	Engvall et al. ^18^	20 (18% women and 26% men)	RC and central obesity were common in patients with RA. Patients with RC displayed reduced FFM together with stable or increased FM, with high levels of LDL, low levels of athero-protective anti-PC and high frequency of hypertension. No difference was recorded in the diet of patients with RC, or not. Low disease activity was associated with a high-SFA diet.
Engvall^18^	Sweden	CS	NR	Karolinska University Hospital	60 patients with RA (83%)	NR	65^M^	ACR	DXA (GE-Lunar Prodigy)	Engvall et al. ^18^	38	A large proportion of inpatients with RA, mainly women, had RC. DAS28, CRP, and IL-6 correlated negatively with LBM.
Papichev^37^	Russia	CS	NR	NR	110 patients with RA (NR%)	NR	52.2±8.1^MSD^	ACR/EULAR	DXA	Engvall et al. ^18^	22.7	The prevalence of RC was the same in patients taking GC (22.2%) and who didn’t (23,7%). Median cumulative dose of oral GC in those with RC was higher, but not significant. Median serum fetuin-A levels were only slightly significantly lower in patients with RC.
Ångström^38^	Sweden	CS	2013–6	Department of Rheumatology, University Hospital of Umeå	87 patients with early RA (NR%)	71.3	60^M^	ACR/EULAR	DXA (GE-Lunar Prodigy)	Engvall et al. ^18^ and Elkan et al*.* ^44^	24 (Engvall) and 32 (Elkan)	RC was associated with reduced aerobic capacity, low-intensity PA, and higher BMI, waist circumference, BW and ESR at the time of diagnosis.
Roubenoff^12 †^	USA	CS	^†^	^†^	24 patients with RA	^†^	^†^	^†^	BIA	^†^	67	LBM was inversely associated with the number of swollen joints. Among the whole participant group, there was a trend toward increasing disability with decreased LBM after adjusting for joint pain and disease duration.
Pineda-Juárez^39^	Mexico	CS	2015–6	Instituto Nacional de Ciencias Médicas y Nutrición Salvador Zubirán and Instituto Nacional de Rehabilitación Luis Guillermo Ibarra Ibarra	224 women with a diagnosis of RA (100%)	NR	52.7±14.2^MSD^	ACR/EULAR	BIA (RJL Quantum X, RJL Systems)	BIVA	51.8	All cachectic subjects, independently of BMI group, had lower levels of serum albumin.
Müller^40^	Estonia	CC	2012–4	Patients: NR Controls: Postal invitations	91 patients with early RA (72%) and 328 healthy controls (54%)	NR	19–79^R^	ACR/EULAR	DXA (Lunar Prodigy Advance)	ALM/height^2^ <20^th^ PC of the sex specific values and BF> 25% for men and >35% for women	26.4	Body composition of patients with recent onset RA differed from that of controls. Approximately 12.4% of women at early stage RA were sarcopenic and overfat (had RC).
Murillo-Saich^42^	Mexico	CC	NR	NR	84 women with RA and 127 healthy women as controls (100%)	NR	24–89^R^	ACR	DXA (Lunar Prodigy Advance)	Engvall et al. ^18^	14.3	No difference was observed in myostatin levels between RA patients with or without RC. It was estimated that 91.7% of patients with RC were sedentary.
Santo^47^	Brazil	Prospective cohort (1 year)	2015–7	Hospital de Clínicas de Porto Alegre, HCPA	90 patients with RA (88.9%)	90	56.5±7.3^MSD^	ACR/EULAR	DXA (GE-Lunar Prodigy Primo)	Engvall et al. ^18^	12^*^	RC was common, classic cachexia was absent. Disease activity and use of DMARD were associated with changes in body composition and physical function, underscoring the importance of aiming for remission when treating RA.

ACR: American College of Rheumatology; ALM: appendicular lean mass (the sum of the lean tissue in the arms and legs); anti-PC: antibodies against phosphorylcholine; BF: body fat; BIA: bioelectrical impedance analysis; BIVA: Bioelectrical impedance vector analysis; BMD: bone mineral density; BMI: body mass index; CC: case-control; CRP: c-reactive protein; CS: cross-sectional; DAS28: disease activity score (28 joints); DMARD: disease-modifying antirheumatic drug; DRACCO: Dudley RA comorbidity cohort; DXA: dual-energy X-Ray absorptiometry; ESR: erythrocyte sedimentation rate; EULAR: European Alliance of Associations for Rheumatology; FFM: mat-free mass; FM: fat mass; GC: glucocorticoid; IL-6: interleukine 6; LBM: lean body mass; LDL: low-density lipoprotein;

^M^median; MS: metabolic syndrome; MSD: mean ± standard deviation; NOD: nod other defined; NR: not reported; PA: physical activity; PC: percentile;

^R^: range; RA: rheumatoid arthritis; RC: rheumatoid cachexia; RR: response rate; SFA: saturated fatty acids; VF: vertebral fractures; ^*^no difference was noted in the prevalence of RC between baseline and the end of the study (at 1 year);

† access to the full-text of the publication was not possible, thus, only available data from the abstract are presented.

### Diagnostic criteria for RC

Diagnostic criteria for RC differed greatly between studies (**Table 2**), with several researchers adopting the Engvall et al.^18^ criteria, some diagnosing RC using the Elkan et al*.*^44^ criteria and others tailoring their own criteria for RC diagnosis.^40^ According to Engvall, 18 RC is diagnosed in patients with RA having a fat-free mass (FFM) below the 10^th^ sex- and ethic-specific percentile and a fat mass index (FMI) exceeding the 25^th^ percentile. The Elkan^44^ criteria appear to be more liberal, diagnosing RC in every patient with RA exhibiting a FFM below the 25^th^ percentile, in parallel to an FMI exceeding the 50^th^ percentile. Ångström and associates^38^ and Hugo et al.^41^ compared the two diagnostic criteria and revealed a greater prevalence of RC when the Elkan^44^ criteria were applied.

**Table 2.** Diagnostic criteria for rheumatoid cachexia used in the literature.

**Diagnosis**		**BW loss** ^†^	**FFMI** (kg/m^2^)	**FMI** (kg/m^2^)	**BF** (% of BW)	**Low muscle strength**	**Fatigue**	**Anorexia**	**Abnormal biochemistry**
	Engvall et al.^18^		<10^th^ PC *	>25^th^ PC^*^					
Rheumatoid cachexia	Elkan et al*.*^44^		<25^th^ PC *	>50^th^ PC^*^					
	Müller^40^		ALM/height^2^ <20^th^ PC *		>25% for men, >35% for women				
Cachexia	Evans et al.^48^	≥5% in the previous 12 months, or less in the presence of illness, or BMI <20 kg/m^2^, **plus**3/5 of the other criteria (on the right columns)	<10^th^ PC*, or ASMI (by DXA) <7.25 in men and <5.45 in women			+ (assessed via handgrip strength)	+	Limited food intake (i.e. EI<20 kcal/kg of BW/day, or <70% of usual food intake), or poor appetite	○ Elevated inflammatory markers (CRP>5.0 mg/L, or IL-6 >4.0 pg/mL) ○ Anaemia (Hb <12 g/dL) ○ Serum albumin <3.2 g/dL

ALM: appendicular lean mass (the sum of the lean tissue in the arms and legs: calculated by DXA); ASMI: appendicle skeletal muscle index; BMI: body mass index; BF: body fat; BW: body weight; CRP: c-reactive protein; DXA: dual-energy x-ray absorptiometry; EI: energy intake; Hb: haemoglobin; FMI: fat mass index; FFMI: fat-free mass index; IL-6: interleukin 6; PC: percentile;

* of the sex-, age-, and ethnic-specific values;

†: oedema free. Background colours denote the component criteria used in each definition.

Müller and associates^40^ adopted their own criteria, diagnosing RC in patients with ALM/height^2^<20^th^ percentile for sex and ethnicity and BF (as a % of BW) >25% for men and >35% for women. Pineda-Juárez^39^ diagnosed RC in their sample using bioelectrical impedance vector analysis (BIVA).

Van Bokhorst and associates^35^ applied the stricter staging system for general cachexia suggested by Evans et al.^48^ These incorporate BW loss (exceeding 5% in the previous 12 months or less in the presence of underlying illness) as the primary criterion, corroborated towards a diagnosis by three of the remaining criteria, namely a) low fat-free mass index (FFMI < 10^th^ percentile), b) low muscle strength, c) fatigue, d) anorexia, and e) abnormal biochemistry regarding CRP, hemoglobin and IL-6 concentrations (Table 2). The researchers concluded that these criteria do not appear to be clinically useful for the identification and diagnosis of RC, mainly due to the low percentage of patients with RA exhibiting BW loss, although they may be of use for other patient groups with frequent cachexia. Consensus criteria for cachexia, defined as severe loss of BW, muscle and fat mass, were developed by the European Society for nutrition and metabolism (ESPEN), but none of the studies on RA patients have applied these criteria.^49^ Furthermore, cachexia is a distinct entity from RC, thus specific diagnostic criteria are required.

Among the methods used to assess body composition in the identified primary studies, DXA was the most popular one, applied in 8 studies,^18,36–38,40–42,44,45,47^ followed by bioelectrical impedance analysis (BIA).^12,35,39,44,45^ Skinfolds were only applied by Lombard et al.,^34^ using the Durnin and Womersley and Siri equations for the calculation of BF and body density, respectively. Elkan and associates^44,45^ additionally compared BIA to DXA and reported that the two methods appear to have a good relative agreement, however, the limits of agreement are wide, indicating that the use of BIA may be restricted for RC diagnosis in clinical practice.

### RC and sex

According to Engvall,^18^ RC appears to affect predominately women, however, the great majority (83%) of participants in his study were women. On the other hand, Table 1 indicates that women consist of the predominant sex in all of the primary studies,^17^ without necessarily demonstrating a greater prevalence of RC.^36,44,45^

It should be kept in mind that RA affects more women than men and that body composition changes during menopause, peri- and post-menopausal status is associated with greater loses in ALM and LBM, in general.^50^ According to the longitudinal Study of Women’s Health Across the Nation (SWAN) cohort, the transition to menopause is associated with a double rate of BF accumulation, whereas the use of hormone replacement therapy (HRT) does not appear to predict changes in body composition independently.^51^ Overall, the transition to menopause and its distinct hormonal changes are considered as an important contributors to predisposing women to sarcopenia and osteoporosis, further aggravating the low physical activity levels.^50^ On the other hand, a recent meta-analysis^52^ suggested that the observed changes in the body composition of women are the result of ageing alone, with menopause possibly contributing only to the decrease in leg FM and the concomitant increase in central fat deposition.

On the flip side, analysis of the University of California San Francisco (UCSF) cohort^53^ suggested that the LBM deficits observed in RA are greater among men, due to testosterone levels. In men, testosterone levels consist of an important regulator of muscle mass, and according to research, testosterone concentrations decrease in men with an RA diagnosis.^54^ Furthermore, improvements in disease activity have been shown to improve testosterone levels acutely, verifying the fact that inflammation suppresses testosterone production in this group.^54–56^ According to Cutolo and Straub^57^, oestrogens act in both enhancing and inhibiting immune reactions, whereas androgens and progesterone exhibit anti-inflammatory and immunosuppressive effects^57,58^ and for this, RA has been suggested to be more severe in men compared to women. Moreover, the phenomenon of androgen-to-oestrogen conversion (intracrinology) is enhanced in inflamed tissues and RA in particular,^59,60^ indicating why androgen concentrations are frequently low, whereas on the other hand, oestrogens levels remain normal in patients with RA.^57^ These changes prompted researchers to propose androgens and progesterone as favourable therapeutic options in RA.^57^

In conclusion, both sexes appear predisposed to altered body composition levels, either as a result of RA (men in particular), or the ageing process and some aspects of the menopause transition (women).

### Disease activity and disease duration

#### Epidemiology

According to Fukuda,^61^ the continuation of inflammation appears to be essential for the decrease in muscle protein to occur in RC. Elevation of disease activity increases BMI through an increased deposit of FM, with a parallel decrease in visceral muscle being observed. As a result, research in Morocco revealed that greater disease activity and duration were associated with the prevalence of RC,^36^ whereas Engvall^18^ suggested that DAS28 correlated negatively with the LBM of patients. Uutela^62^ further verified this, by showing that poorer muscle performance composite score (MPCS) was associated with greater disease activity in patients with RA. Further evidence was also provided early on by Roubenoff,^12^ who reported that LBM was associated with the number of swollen joints and a more recent study showing that disease activity was associated with changes in body composition, indicating the importance of aiming for remission when treating RA.^47^ On the other hand, the greatly heterogenous pooled data by Santo^17^ failed to reveal the existence of a relationship between disease activity and duration with the prevalence of RC, in line with the results of Santillán-Díaz’s^22^ underpowered study. Thus, it is logical to assume that the lack of a relationship between disease activity and RC in these studies is probably the result of lower methodological quality.

#### Interventions

Since proinflammatory cytokines, and particularly TNF-α, are important inducers of RC,^13,63^ it is logical to expect that a reduction in inflammation (including TNF-α), would attenuate RC.^5^ However, a treat-to-target therapeutic approach with tight control in RA failed to attenuate RC.^5^ According to Lemmey, 5 a possible explanation is that RC occurs early in the course of RA, probably even during the preclinical phase. This is indicated by the fact that muscle atrophy and decline in muscle strength are already present before the onset of pain and swelling in patients with RA.^64^ In the same vein, RC was associated with erythrocyte sedimentation rate (ESR) at the time of diagnosis^38^ and a similar degree of muscle depletion in patients with early RA,^6,65^ as well as in those with established RA.^66^ Nevertheless, BF appears to accumulate with disease duration,^6^ as a possible result of therapy.

### Comorbidities associated with RC

Reported comorbidities associated with RC in relevant research include hypertension,^44,45^ excess in body weight (overweight and obesity).^34–36^ However, it should be noted that RC does not appear to increase cardiovascular disease (CVD) risk.^19^ According to Masuko, the exact role of RC in increasing cardiovascular risk and clinical prognosis in RA is not clearly understood.^67^ However, when patients with RC and RC-free ones were compared, no differences were observed regarding classical or novel risk factors for cardiovascular disease (CVD), the prevalence of established CVD, or 10-year CVD mortality risk.^38,43,67^ According to Elkan, 68 low physical activity levels in RA are associated with increased CVD risk, irrespective of body composition.

In an early case-control study of patients with RC, muscle density decline was associated with numerous inflammatory factors.^69^ RA disability was associated with adverse changes in body composition, with health assessment being inversely related to the ALM.^70^ However, RC and disability have only been evaluated by Roubenoff and associates,^12^ in the first recorded study evaluating the prevalence of RC. No other research has evaluated RC and disability together thus far. Despite the lack of research identifying comorbidities in RC, given that cachexia is a major factor for increased mortality risk, prompt interventions are required to prevent fatal metabolic abnormalities.^67^

### Rheumatic drugs, body composition and RC

**Table 3** details all primary studies investigating changes in body composition parameters in patients with RA, following individual medication interventions, or combination therapeutic schemes. Methotrexate (MTX) monotherapy is the first line DMARD agent for DMARD-naïve patients with RA.^71,72^ It is yet unknown if MTX monotherapy for RA,^71,72^ is beneficial for RC.^67^ Santillán-Díaz^22^ and associates suggested that MTX administration with concomitant folic acid (FA) supplementation may protect against the development of RC. FA is a known cofactor in the metabolism of homocysteine, and low homocysteine levels are associated with greater muscle degradation, reduced muscle strength and lower physical function.^73,74^ On the other hand, oral nutrient supplementation (ONS) with FA has been shown to reduce homocysteine concentrations during MTX therapy.^75^

**Table 3.** Primary studies evaluating the effects of medication for RA on body composition and cachexia.

**First author**	**Design**	**Participants**	**Intervention(s)**	**Comparator(s)**	**duration**	**Outcomes**	**Results**
Marcora^65^	Parallel RCT	N=26 patients with early RA	ETA (vials reconstituted with bacteriostatic water, injected subcutaneously twice/week) (*n*=12 )	MTX *per os* (7.5 mg/week for a month, increased to a maximum of 15 mg/week for the 2^nd^month and 20 mg/week for the subsequent 4^th^ month if deemed necessary ) (*n*=12 )	24 weeks	Body composition, physical function, disease activity, systemic inflammation, IGF levels	Approximately 44% of BW gained in the etanercept group was FFM, as compared to only 14% in the MTX arm.
Tournadre^83^	CC	N=21 patients with active RA and 21 RA- free matched controls	TCZ (*n*=21 )	No treatment (*n*=21 )	1 year	WC, BMI, BP, lipid profile, FPG, insulin, serum levels of adipokines and pancreatic/gastrointestinal hormones, and body composition (DXA)	Compared with controls, body composition was altered in RA with a decrease in total and appendicular LM, without any changes in BF. Among patients with RA, 28.6% had a skeletal muscle mass index below the cut-off for sarcopenia (4.8% of controls). After 1 year of treatment, a gain in BW was noted, without any changes in FM. An increase in LM was observed with a significant gain in appendicular LM and skeletal muscle mass index between 6–12 months of treatment. FM was decreased in the trunk/peripheral fat ratio, and increased in the subcutaneous adipose tissue. No changes were noted for WC, BP, FPG or atherogenic index.
Schultz^85^	CT	N=11 non-diabetic patients with RD	TCZ (NOD) (*n*=11 )	None	3 months	HOMA-IR, serum levels for leptin, adiponectin, TG, LDL, HDL and Lp(a)	The HOMA-IR decreased significantly, leptin concentrations were not altered but adiponectin levels increased. Serum TG, LDL and HDL tended to be increased, whereas Lp(a) levels was lowered.
Toussirot^84^	Open label CT	N=77 patients with active RA	TCZ IV 8 mg/kg monthly, as administered in daily practice, with the option to decrease TCZ dosage to 4 mg/kg at the rheumatologist’s discretion (*n*=77 )	None	1 year	BMI and anthropometry, lipid and metabolic parameters, serum adiponectin, leptin, resistin, ghrelin, body composition (DXA)	TCZ treatment was associated with an increase in adiponectin, especially at the onset of the treatment and induced a significant gain in LM, BMI and WC, while FM did not change. In addition, TCZ may have an anabolic impact on lean mass/skeletal muscle.
Fioravanti^86^	CT	N=44 patients with active RA	IV TCZ (8 mg/kg) once every 4 weeks (*n*=20 )	IV TCZ (8 mg/kg) once every 4 weeks, plus MTX (*n*=24 )	6 months	BMI, DAS28, HAQ	ESR, CRP, DAS28-ESR and HAQ improved in both arms. TC was increased and chemerin was decreased in both arms.
Chen^76^	Non-randomized CT	N=30 patients with RA	Subcutaneous injections of ETA twice weekly (*n*=20 )	Non-biological DMARDs (*n*=10 )	12 months	BW, BF, appetite rating, lipid profiles, gut hormones and leptin	ETA induced significant BW gain, hyperuricemia, decreased fasting plasma GIP levels, and loss of post-oral glucose suppression of plasma leptin concentration. Appetite score and serum lipid profiles did not change.
Toussirot^79^	Parallel CT	N=8 patients with RA non-responders to DMARDs and 12 with AS	IFX (3 or 5 mg/kg in RA and AS, respectively) (*n*=3 )	- ETA (50 mg/weekly) (*n*=7 ) - ADA (40 mg every other week) (*n*=12 )	2 years	DXA, HAQ, ESR, CRP, DAS28, serum leptin, adiponectin, resistin, and ghrelin levels, body composition (DXA)	A gain in BMI and a tendency for BW gain, android and visceral fat increase was noted in patients with RA.
Serelis^78^	Parallel CT	N=19 women with active RA	IFX (3 mg/kg) at weeks 0, 2, 6 and thereafter systematically every 8 weeks (*n*=10 )	ADA (standard dose of 40 mg subcutaneously every 14 days) (*n*=9 )	1 year	BMI, body composition (DXA), adiponectin	No change in LBM, or FM and no difference was noted in lumbar spine BMD. Serum concentrations of adiponectin increased after 1 year of anti-TNF treatment.
Engvall^77^	Parallel RCT	N=40 patients with early RA who failed MTX treatment (20 mg/week for 3 months)	MTX (20 mg/week) plus SSZ and hydroxychloroquine (*n*=22 )	MTX treatment (20 mg/week) plus IFX (*n*=18 )	21 months	Body composition (DXA), BMD, leptin, adiponectin, apolipoproteins, IGF-1	Patients treated with anti-TNF had a significant increase in fat mass compared to the other arm, despite similar reduction in disease activity. Both treatments prevented loss of muscle mass and bone. Leptin concentrations increased in both arms. No changes were recorded for apolipoproteins or IGF-1. The markers of bone resorption decreased at 12 months in both arms, without difference between them.
Konijn^30^	Parallel RCT	N=108 prednisolone-naive patients with recent-onset RA	COBRA (prednisolone 60 mg/day, tapered to 7.5 mg/day in 6 weeks; MTX and SSZ) (*n*=54 )	COBRA-light therapy (PSL 30 mg/day, tapered to 7.5 mg/day in 8 weeks, plus MTX) (*n*=54 )	26 weeks	Body composition (DXA)	There were no differences between the treatment groups. BW and FM were increased. The trunk/peripheral fat ratio and the proportional distribution of BW and FM remained stable over time.
Metsios^24^	CC	N=20 patients with RA and N=12 healthy controls	anti-TNF treatment (*n*=20 )	No treatment (*n*=12 )	12 weeks	BIA (Tanita BC-418-MA), TNF-α, physical activity, REE, DAS28	There were no significant changes in FFM or BF. ESR, DAS28, HAQ, and TNF-α improved.

ADA: adalimumab; AS: ankylosing spondylitis; BF: body fat; BIS: bioelectrical impedance spectroscopy; BMD: bone mineral density; BMI: body mass index; BP: blood pressure; BW: body weight; CC: case-control; COBRA: Combinatietherapie bij Reumatoide Artritis^118^; CT: clinical trial; DAS28: disease activity score (28 joints); DMARD: disease-modifying antirheumatic drug; DXA: dual x-ray absorptiometry; ESR: erythrocyte sedimentation rate; ETA: etanercept; FFM: fat-free mass; FM: fat mass; FPG: fasting plasma glucose; GIP: glucose-dependent insulinotropic polypeptide; HAQ: health assessment questionnaire; HDL: high-density lipoprotein; HOMA-IR: homeostatic model for insulin resistance; IFX: infliximab; IGF: insulin-like growth factor; IV: intravenous; LDL: low-density lipoprotein; LM: lean mass; Lp(a): lipoprotein (a); MTX: methotrexate; NOD: not other defined; PSL: prednisolone; RA: rheumatoid arthritis; RCT: randomized controlled trial; RD: rheumatic diseases; REE: resting energy expenditure; SSZ: sulfasalazine; TBP: total body protein; TC: total cholesterol; TCZ: tocilizumab; TG: triglycerides; TNF: tumor necrosis factor; WC: waist circumference.

In a comparative effectiveness study, Marcora and associates^65^ compared the use of TNF blocking agents (etanercept, ETA) to MTX among patients with RA and showed that the latter induced a 14% increase in FFM compared to a 44% increase observed in the ETA arm. Both therapies were effective in controlling disease activity and improving physical function. Chen and associates^76^ compared subcutaneous injections of ETA against non-biological DMARDs for 1 year and observed a greater BW gain, hyperuricemia prevalence, decreased fasting plasma glucose-dependent insulinotropic polypeptide (GIP) concentrations, and loss of post-oral glucose suppression of plasma leptin concentration in the ETA arm. In a similar trial, Engvall^77^ compared treatment with a combination of DMARDs against MTX plus anti-TNF (infliximab, IFX) for a total of 2 years. IFX therapy induced an increment in BF mass, whereas a similar effect was not achieved with the DMARDs combination, despite the fact that both treatments resulted in akin reductions in disease activity. This indicated that the accumulation of FM appears to be drug-specific. In a small sample of women with an RA diagnosis, Serelis^78^ evaluated the effect of IFX compared to adalimumab (ADA), on the body composition and adiponectin levels of participants. One year of intervention failed to induce any changes in LBM and FM and no differences were noted in the lumbar spine BMD of participants. On the other hand, anti-TNF treatment for 1-year increased serum adiponectin concentrations significantly. In a similar trial, Toussirot^79^ administered three different anti-TNF agents (IFX, ETA, or ADA) to patients with RA and ankylosing spondylitis (AS). After two years of follow-up, a gain in BMI was recorded and an increase in visceral and android fat among patients with RA. Collectively these trials suggest that anti-TNF agents are not effective in increasing muscle mass and are associated with greater FM as compared to csDMARD alone, in particular regarding fat accumulated in the trunk.^24,65^

However, an epidemiological analysis of the Veterans Affairs RA (VARA) registry^80^ revealed that MTX was associated with a reduced risk of BW loss, whereas prednisone or anti-TNF therapies were not associated with changes in the BMI or the risk of BW loss, independent of other factors. According to Santillán-Díaz,^22^ this MTX-induced positive metabolic balance can explain its effects on the immune response effects, namely the extracellular increase in adenosine, and the inhibition of the transcription factor NFκB, the endothelial and inflammatory cell function, the T-cell function regulation.^81,82^

Prednisolone, is a GC, reducing inflammation and improving physical function rapidly and effectively, thus, frequently used as a first-line treatment in RA as part of a combination therapy with other DMARDs. Konijn et al.^30^ investigated the effect of two different prednisolone doses, a high-dose and a step-down regimen on the body composition of patients with early RA. Both prednisolone regimens increased total body mass, through an accumulation of FM. However, fat redistribution from peripheral to central tissues was not recorded, contradicting the previous assumption of rapid adverse effects of prednisolone on the body composition of patients with RA. On the other hand, Lin et al*.*^33^ revealed that when patients with a normal BMI were considered, approximately 18.2% exhibited myopenia overlapping with overfat. Moreover, in this normoweight subgroup of patients with RC, the worst radiographic scores and highest rates of previous GC treatment and hypertension diagnosis were also exhibited.^33^ Compared to the patients not exhibiting myopenia and overfat, the normoweight ones with RA on previous GC treatment exhibited a higher rate of myopenia, overlapping with overfat.^33^ In concert to this observation, Hugo and associates^41^ noted that low levels of physical activity and treatment with GCs was associated with increased nutritional complications among patients with RA, including RC and metabolic syndrome. A more recent study conducted in Russia^37^ suggested that the median cumulative dose of oral GC in patients with RC appears to be higher, although the finding was not statistically significant. Nevertheless, an early study^36^ had also reported a greater steroid cumulative dose among patients with RC, indicating that changes in body composition are also therapy-driven.

Apart from TNF-α blockers, other anti-inflammatory agents are often employed in the management of RA. Tournadre and associates^83^ evaluated the effect of TCZ treatment, a humanized anti-IL-6 receptor monoclonal antibody, on the body composition and metabolic profile of patients with RA. After one year of treatment with TCZ, BW was significantly increased without any observed changes in the FM. Between months 6–12 of treatment, an increase in appendicular lean mass and skeletal muscle mass index was observed, with a redistribution of BF. The trunk/peripheral fat ratio was decreased and subcutaneous adipose tissue was increased. No changes were noted regarding blood pressure, waist circumference, fasting glucose concentrations, or the atherogenic index of participants. These findings were also confirmed by a French multicentric study, the évolution *des ADIpokines et de la composition corporelle chez les patients atteints de Polyarthrite Rhumatoïde et recevant un traitement par Tocilizumab* (ADIPRAT) phase IV open-label clinical trial.^84^ In the ADIPRAT,^84^ patients with RA were administered intravenous TCZ (8 mg/kg monthly), as administered in daily practice, with the option to decrease TCZ dosage to 4 mg/kg at the rheumatologist’s discretion and the possibility to reduce GCs intake whenever deemed required. After one year of intervention, an increase in adiponectin concentrations was noted, especially at the onset of the treatment. Furthermore, TCZ induced significant gain in LM, BMI and waist circumference, while FM remained unchanged. Schultz and associates^85^ evaluated the administration of TCZ in 11 patients with rheumatic diseases (no further information was provided), who were diabetes-free. TCZ treatment for 3 months significantly decreased the homeostatic model for insulin resistance (HOMA-IR), and increased serum adiponectin concentrations. Serum triglycerides, LDL and HDL tended to be increased, whereas lipoprotein (a) levels were lowered post-treatment.^85^ Finally, Fioravanti^86^ evaluated TCZ administration (8 mg/kg TCZ IV, once every 4 weeks) as monotherapy, or on top of MTX treatment for a total 6 months. ESR, CRP, DAS28-ESR and health assessment questionnaire (HAQ) were improved in both arms. In parallel, total cholesterol concentrations were increased and chemerin was decreased in both arms.

Furthermore, a variety of methodological issues are apparent in research. Overall, it is difficult to define the exact effect medications have on RC, as most of the studies are either comparing different medication regimens, as it is unethical to leave the patients without medication. Moreover, several trials^83–85^ used control arms comprising of patients not receiving any medication, whereas one trial used healthy controls as comparators^24^. With regard to the participants included in the aforementioned research items, the majority of studies used patients with RA, medication-naïve or on medication, with active or inactive RA, where changes in body composition were recorded during the course of intervention. Only the trial conducted by Schultz^85^ used patients with RC as the patient pool for their intervention.

### Dietary treatments, body composition and RC

Observational data from the National Health and Nutrition Examination Survey (NHANES) and several smaller studies indicate that patients with RA tend to follow diets of low or suboptimal quality.^87–92^ Medical nutrition therapy (MNT) in RA mainly aims in stimulating an overexpression of anti-inflammatory cytokines^93^ and controlling RC, with tailored nutrition interventions focusing on overcoming impaired protein synthesis and reverting muscle catabolism. A total of four intervention studies have evaluated the use of ONS in body composition among patients with RA, using an RCT design (**Table 4**). Wilkinson^94^ administered ONS against placebo as an add-on therapy for 12 weeks and revealed that although supplementation with creatine increased total and appendicular LM, it failed to improve isometric knee extensor and handgrip strength, and physical function. Marcora and associates^95^ compared the results of two different amino-acid drinks administered for 12 weeks, one containing 3 g beta-hydroxy-beta-methylbutyrate (HMB, calcium salt), 14 g of L-arginine (ARG) and 14 g L-glutamine (GLN), diluted in 240 mL of water, drank twice/daily and a comparator drinks with a nitrogen (7.19 g/day) and calorie (180 kcal/day) balanced mixture of ARG (11 g), GLN (1.75 g), L-glycine (6.10 g) and L-serine (4.22 g), diluted in 240 mL of water, drank twice/daily. Dietary supplementation with HMB/GLN/ARG was not superior to placebo in the treatment of RC. However, both amino acid mixtures increased FFM, total body protein (TBP), arms and legs LM, and some measures of physical function.

**Table 4.** RCTs evaluating effects of oral nutrient supplementation on body composition and cachexia among patients with RA.

**First author**	**Design**	**Participants**	**Intervention(s)**	**Comparator(s)**	**duration**	**Outcomes**	**Results**
Wilkinson^94^	Parallel RCT	N=40 patients with RA	Creatine ONS [20 g of Cr monohydrate (MyProtein, 4×5 g/day for the initial 5 days (loading dose) followed by 3 g/day for the remainder of the 12-week period (maintenance dose)], mixed with a mango-flavoured drink powder (Foster Clark Products) to improve taste (*n*=15 )	Placebo ONS (flavoured drink powder) (*n*=20 )	12 weeks	Body composition (DXA and BIS), strength, aerobic capacity (Vo_2max_) and physical function	ONS with creatine increased total and appendicular LM, but failed to improve isometric knee extensor and handgrip strength, or objectively assessed physical function.
Marcora^95^	Parallel RCT	N=40 patients with RA	ONS with 3 g HMB (calcium salt), 14 g ARG and 14 g GLN, diluted in 240 mL of water, drank twice/daily (*n*=20 )	ONS with placebo (nitrogen (7.19 g/day) and calorie (180 kcal/day) balanced mixture of ARG (11 g), GLN (1.75 g), L-glycine (6.10 g) and L-serine (4.22 g), diluted in 240 mL of water, drank twice/daily (*n*=20 )	12 weeks	Body composition and physical function	Dietary ONS with HMB/GLN/ARG was not superior to placebo in the treatment of RC. Both amino acid mixtures increased FFM, TBP, arms and legs LM, and some measures of physical function.
Lovell^97^	Parallel RCT	N=198 children and adolescents with juvenile RA	ONS with 1,000 mg of Ca and 400 IU of vitamin D (*n*=103 )	ONS with matched placebo tablets and 400 IU of vitamin D (*n*=95 )	24 months	BMD (DXA)	At 24 months, the mean BMD among those receiving Ca was greater, independently of sex, Tanner stage, adherence to medication, etc.
Aryaeian^96^	Parallel RCT	N=62 patients with active RA	ONS with 6 capsules of 500 mg barberry extract (*n*=31 )	ONS with placebo (HPMC) (*n*=31 )	3 months	FPG, TG, LDL and HDL levels, BMI, SBP, DBP, anthropometry	BW, BMI, and conicity index increased in both groups, but this was significant only in the placebo group. BF (% of BW), LDL, hips circumference, FPG, and SBP were all decreased in the intervention arm.

ARG: L-arginine; BF: body fat; BIS: bioelectrical impedance spectroscopy; BMD: bone mineral density; BMI: body mass index; BW: body weight; DAS28: disease activity score (28 joints); DBP: diastolic blood pressure; DXA: dual x-ray absorptiometry; ESR: erythrocyte sedimentation rate; FFM: fat-free mass; FM: fat mass; FPG: fasting plasma glucose; GLN: L-glutamine; HDL: high-density lipoprotein; HMB: beta-hydroxy-beta-methylbutyrate; HPMC: hydroxypropyl methylcellulose; IGF: insulin-like growth factor; LDL: low-density lipoprotein; LM: lean mass; ONS: oral nutrient supplementation; RA: rheumatoid arthritis; RC: rheumatoid cachexia; RCT: randomized controlled trial; SBP: systolic blood pressure; TBP: total body protein; TG: triglycerides.

Aryaeian et al.^96^ hypothesised that ONS with barberry extract might improve anthropometry and glycemia in patients with RA. Using a placebo-controlled design, they demonstrated that after 3 months BW, BMI, and conicity index increased similarly in both groups. On the other hand, BF (%), low-density lipoprotein (LDL), hips circumference, fasting plasma glucose (FPG), and systolic blood pressure were all decreased in the intervention arm compared to the placebo.

Lovell^97^ evaluated supplemental Calcium (Ca) intake on the bone mineral density (BMD) of patients with juvenile RA. The two treatment arms received 1,000 mg of Ca and 400 IU of vitamin D, or vitamin D alone, for a duration of two years. At 24 months, the mean BMD among the youngsters receiving Ca was greater, independently of sex, Tanner’s puberty stage, medication adherence, or other factors. Finally, recent research on animals indicated that ONS with coriander^98^ might have favorable effects in restoring muscle mass, however, this finding has not been verified on humans.

In summary, with regard to the dietary supplements, amino acids appear to be promising in improving body composition and possibly attenuating RC although more research is required to validate the findings. Antioxidant supplements like barberry extract appear to target glycemia and by inference, an improvement in other metabolic factors, including anthropometry, may be noted.

### Metabolism, body composition and RC

According to Spies,^99^ in RA, energy metabolism is regulated by chronic inflammation, not only with regard to energy supply, but additionally for immune response activation and control, through a variety of metabolic signals.^100,101^ Thus, the existence of chronic, low-grade inflammation diverts energy and glucose metabolism from other systems in order to support the requirements for immune response activation.^102,103^ In the presence of a chronic pro-inflammatory environment, macrophages and lymphocytes switch from a resting state to a highly active one, pedaling the release of host-defense factors promoting phagocytosis and antigen production.^104^ To counterbalance this metabolic shift, lipid and protein catabolism increase for the production of ATP.^102^ The higher inflammatory cytokine production stimulates muscle catabolism^10^ through the nuclear-factor κB (NK-κB)-dependent pathway,^105^ increasing the whole-body protein turnover, triggering muscle loss and the development of cachexia.^106^ This net protein shortage further increases metabolic rate and in particular, resting energy expenditure (REE) in patients with RA.^12,13,24,107^ However, total energy expenditure (TEE) levels appear lower compared to the rest of the population, mainly as a result of low physical activity levels.^108^

TEE consists of the sum of the thermic effect of food (TEF), REE and physical-activity energy expenditure (PAEE). With the TEF being dependent on the amount of consumed food, and TEE in RA appears to be mostly influenced by the REE and PAEE of patients. In this manner, the increased REE and the reduced PAEE are developing an energy deficiency environment, further driving the cachectic state.^109^

Several lines of evidence suggest that REE, disease activity and severity are inter-related in RA and RC. In all of these studies, REE was measured using indirect calorimetry.^107,110,111^ When REE is calculated using prediction equations instead of being measured, it is not associated with any score or biomarker of disease activity.^112^ On the other hand, RA-specific equations for the calculation of REE have been proposed, taking into account the levels of CRP.^113^

In the same vein, although malnutrition would be the logical sequence in this inflammatory environment, the use of standard malnutrition assessment tools seems to fail to detect the need for nutrition intervention in RA, as there may be an increase in FM.^45,88,114^ This results in masking the identification of patients in higher risk for RC, since the assessment of cachexia is rarely performed in clinical practice.

### Limitations of existing research

A major limitation in the available research on RC involves the diagnostic criteria applied. RA sensitive criteria are necessary for RC diagnosis although it appears that the use of different criteria, not specific to RA increase bias and do not allow for generalization or pooling of the primary studies. Thus, it appears that the need for conducting studies of better quality aiming to assess the prevalence of RC is apparent.

## CONCLUSIONS

In 1873, Sir James Paget^115^ first described RC; however, research on effective interventions to manage this important problem remains in infancy. It appears that the complex pathophysiology of RA, the adverse effects of polypharmacy in body composition, and the deteriorating metabolic alterations, develop a complex situation which becomes difficult to balance. Nonetheless, it appears that triage for RC identification should be performed as early as RA diagnosis, with constant monitoring of the condition thereafter, in order to better understand the mechanistic effects of RC and improve prognosis in affected patients.

## References

[1] Torpy JM Perazza GD Golub RM. Rheumatoid Arthritis. JAMA 2011;305:1824–1824. 21540429 10.1001/jama.305.17.1824

[2] Almutairi K Nossent J Preen D Keen HI Inderjeeth CA. The global prevalence of rheumatoid arthritis: a meta-analysis based on a systematic review. Rheumatol Int 2021;41:863–77. 33175207 10.1007/s00296-020-04731-0

[3] Almutairi K Nossent J Preen D Keen H Inderjeeth C. SAT0576 The prevalence of rheumatoid arthitis:a systematic review of population-based studies. Ann Rheum Dis 2020;79:1246–1247.

[4] Turk SA Van Schaardenburg D Boers M de Boer S Fokker C Lems WF An unfavorable body composition is common in early arthritis patients:A case control study. PLoS One 2018;13:e0193377. 29565986 10.1371/journal.pone.0193377 PMC5863963

[5] Lemmey AB. Rheumatoid cachexia:the undiagnosed, untreated key to restoring physical function in rheumatoid arthritis patients? Rheumatology 2016;55:1149–150. 26672906 10.1093/rheumatology/kev412

[6] Book C Karlsson MK Åkesson K Jacobsson LT. Early rheumatoid arthritis and body composition. Rheumatology 2009;48:1128–132. 19602478 10.1093/rheumatology/kep165

[7] Gabdulina G Tokarev A Mussabaeva D. AB0347 Body composition in patients with rheumatoid arthritis kazakh nationality. Ann Rheum Dis 2018;77:1346–7.

[8] Toroptsova N Dobrovolskaya O Demin N Shornikova L. AB0267 Body composition in patients with rheumatoid arthritis. Ann Rheum Dis 2020;79:1432.32883653

[9] Westhovens R Nijs J Taelman V Dequeker J. Body composition in rheumatoid arthritis. Br J Rheumatol 1997;36:444–8. 9159537 10.1093/rheumatology/36.4.444

[10] Rall LC Roubenoff R. Rheumatoid cachexia:metabolic abnormalities, mechanisms and interventions. Rheumatology 2004;43:1219–23. 15292530 10.1093/rheumatology/keh321

[11] Moore FD. Energy and the maintenance of the body cell mass. JPEN J Parenter Enteral Nutr 1980;4:228–60. 6772804 10.1177/014860718000400302

[12] Roubenoff R Roubenoff RA Ward LM Holland SM Hellmann DB. Rheumatoid cachexia: depletion of lean body mass in rheumatoid arthritis. Possible association with tumor necrosis factor. J Rheumatol 1992;19:1505–10. 1464859

[13] Roubenoff R Roubenoff RA Cannon JG Kehayias JJ Zhuang H Dawson-Hughes B Rheumatoid cachexia: cytokine-driven hypermetabolism accompanying reduced body cell mass in chronic inflammation. J Clin Invest 1994;93:2379–86. 8200971 10.1172/JCI117244 PMC294444

[14] Dewys WD Begg C Lavin PT Band PR Bennett JM Bertino JR Prognostic effect of weight loss prior to chemotherapy in cancer patients. Eastern Cooperative Oncology Group. Am J Med 1980;69:491–7. 7424938 10.1016/s0149-2918(05)80001-3

[15] Kotler DP Tierney AR Wang J Pierson RN Jr. Magnitude of body-cell-mass depletion and the timing of death from wasting in AIDS. Am J Clin Nutr 1989;50:444–7. 2773823 10.1093/ajcn/50.3.444

[16] Metsios GS Stavropoulos-Kalinoglou A Koutedakis Y Kitas GD. Rheumatoid Cachexia: causes, significance and possible interventions. Hosp Chronicles 2006;1:20–2.

[17] Santo RCE Fernandes KZ Lora PS Filippin LI Xavier RM. Prevalence of rheumatoid cachexia in rheumatoid arthritis:a systematic review and meta-analysis. J Cachexia Sarcopenia Muscle 2018;9:816–25. 30133186 10.1002/jcsm.12320 PMC6204596

[18] Engvall I-L Elkan A-C Tengstrand B Cederholm T Brismar K Hafstrom I. Cachexia in rheumatoid arthritis is associated with inflammatory activity, physical disability, and low bioavailable insulin-like growth factor. Scand J Rheumatol 2008;37:321–8. 18666027 10.1080/03009740802055984

[19] Summers GD Metsios GS Stavropoulos-Kalinoglou A Kitas GD. Rheumatoid cachexia and cardiovascular disease. Nat Rev Rheumatol 2010 68 2010;6:445–1. 10.1038/nrrheum.2010.105 20647995

[20] Stavropoulos-Kalinoglou A Metsios GS Koutedakis Y Nevill AM Douglas KM Jamurtas A Redefining overweight and obesity in rheumatoid arthritis patients. Ann Rheum Dis 2007;66:1316–21. 17289757 10.1136/ard.2006.060319 PMC1994320

[21] Walsmith J Abad L Kehayias J Roubenoff R. Tumor necrosis factor-alpha production is associated with less body cell mass in women with rheumatoid arthritis. J Rheumatol 2004;31:23–9. 14705214

[22] Santillán-Díaz C Ramírez-Sánchez N Espinosa-Morales R Orea-Tejeda A Llorente L Rodríguez-Guevara G Prevalence of rheumatoid cachexia assessed by bioelectrical impedance vector analysis and its relation with physical function. Clin Rheumatol 2018;37:607–14. 29119481 10.1007/s10067-017-3889-x

[23] Tierney M Fraser A Kennedy N. Physical activity in rheumatoid arthritis:a systematic review. J Phys Act Health 2012;9:1036–48. 22971883 10.1123/jpah.9.7.1036

[24] Metsios GS Stavropoulos-Kalinoglou A Douglas KMJ Koutedakis Y Nevill AM Panoulas VF Blockade of tumour necrosis factor-alpha in rheumatoid arthritis:effects on components of rheumatoid cachexia. Rheumatology (Oxford) 2007;46:1824–7. 18032540 10.1093/rheumatology/kem291

[25] Fenton SAM van Zanten JJCSV Duda JL Metsios GS Kitas GD. Sedentary behaviour in rheumatoid arthritis:definition, measurement and implications for health. Rheumatology (Oxford) 2018;57:213–26. 28398519 10.1093/rheumatology/kex053

[26] Bravo Ramírez A Hurtado Torres GF Martínez Martínez M Abud Mendoza C. Análisis de la composición corporal en mujeres adultas con lupus eritematoso sistémico. Nutr Hosp 2012;27:950–951. 23114961 10.4321/S0212-16112012000300032

[27] Tengstrand B Carlström K Hafström I. Bioavailable testosterone in men with rheumatoid arthritis—high frequency of hypogonadism. Rheumatology 2002;41:285–289. 11934965 10.1093/rheumatology/41.3.285

[28] Baillargeon J Al Snih S Raji MA Urban RJ Sharma G Sheffield-Moore M Hypogonadism and the risk of rheumatic autoimmune disease. Clin Rheumatol 2016;35:2987. 10.1007/s10067-016-3330-x PMC5544431 27325124

[29] Letarouilly J-G Flipo R-M Cortet B Tournadre A Paccou J. Body composition in patients with rheumatoid arthritis: a narrative literature review. Ther Adv Musculoskelet Dis 2021;13:1759720X211015006. 10.1177/1759720X211015006 PMC8221676 34221129

[30] Konijn NPC van Tuyl LHD Boers M van de Ven PM den Uyl D Ter Wee MM The short-term effects of two high-dose, step-down prednisolone regimens on body composition in early rheumatoid arthritis. Rheumatology (Oxford) 2016;55:1615–22. 27247434 10.1093/rheumatology/kew221

[31] Giles JT Ling SM Ferrucci L Bartlett SJ Andersen RE Towns M Abnormal body composition phenotypes in older rheumatoid arthritis patients:association with disease characteristics and pharmacotherapies. Arthritis Rheum 2008;59:807–15. 18512711 10.1002/art.23719 PMC2670994

[32] Ekici R Erden A Güven SC Armağan B Özdemir B Karakaş Ö Prevalence of sarcopenia and clinical implications in patients with newly diagnosed rheumatoid arthritis. Nutrition 2021;90:111353. 34192633 10.1016/j.nut.2021.111353

[33] Lin J-Z Chen C-T Ma J-D Mo YQ Li QH Chen LF Neglected extra-articular manifestations in rheumatoid arthritis patients with normal body mass index:reduced skeletal muscle overlapping overfat. Ther Adv Chronic Dis 2020;11:204062232097524. 10.1177/2040622320975241 PMC7705189 33294150

[34] Lombard LA du Plessis LM Visser J. Body composition of rheumatoid arthritis patients in the City of Cape Town, South Africa. Clin Rheumatol 2014;33:467–76. 24196988 10.1007/s10067-013-2414-0

[35] van Bokhorst - de van der Schueren MAE Konijn NPC Bultink IEM Lems WF Earthman CP van Tuyl LH. Relevance of the new pre-cachexia and cachexia definitions for patients with rheumatoid arthritis. Clin Nutr 2012;31:1008–10. 22695407 10.1016/j.clnu.2012.05.012

[36] El Maghraoui A Sadni S Rezqi A Bezza A Achemlal L Mounach A. Does Rheumatoid Cachexia Predispose Patients with Rheumatoid Arthritis to Osteoporosis and Vertebral Fractures? J Rheumatol 2015;42:1556–62. 26233497 10.3899/jrheum.141629

[37] Papichev E Zavodovsky B Seewordova L Polyakova J Akhverdyan Y. POS0509 Prevalence of rheumatoid cachexia and its association with serum fetuin-a levels in caucasian patients with rheumatoid arthritis. Ann Rheum Dis 2021;80:487–488.33451999

[38] Ångström L Hörnberg K Sundström B Södergren A. Rheumatoid cachexia in early rheumatoid arthritis: prevalence and associated variables. Scand J Rheumatol. Epub ahead of print 2021. 10.1080/03009742.2021.1973678 34643160

[39] Pineda-Juárez JA Lozada-Mellado M Ogata-Medel M Hinojosa-Azaola A Santillán-Díaz C Llorente L Body composition evaluated by body mass index and bioelectrical impedance vector analysis in women with rheumatoid arthritis. Nutrition 2018;53:49–53. 29655777 10.1016/j.nut.2018.01.004

[40] Müller R Kull M Põlluste K Valner A Lember M Kallikorm R. Factors Associated With Low Lean Mass in Early Rheumatoid Arthritis: A Cross-Sectional Study. Medicina (Kaunas);55. Epub ahead of print 8 November 2019. 31717450 10.3390/medicina55110730 PMC6915666

[41] Hugo M Mehsen-Cetre N Pierreisnard A Schaeverbeke T Gin H Rigalleau V. Energy expenditure and nutritional complications of metabolic syndrome and rheumatoid cachexia in rheumatoid arthritis:an observational study using calorimetry and actimetry. Rheumatology (Oxford) 2016;55:1202–9. 27009826 10.1093/rheumatology/kew038

[42] Murillo-Saich JD Vazquez-Villegas ML Ramirez-Villafaña M Saldaña-Cruz AM Aceves-Aceves JA Gonzalez-Lopez L Association of myostatin, a cytokine released by muscle, with inflammation in rheumatoid arthritis:A cross-sectional study. Medicine (Baltimore) 2021;100:e24186. 33546034 10.1097/MD.0000000000024186 PMC7837870

[43] Metsios GS Stavropoulos-Kalinoglou A Panoulas VF Sandoo A Toms TE Nevill AM Rheumatoid cachexia and cardiovascular disease. Clin Exp Rheumatol 2009;27:985–988. 20149317

[44] Elkan A-C Håkansson N Frostegård J Cederholm T Hafström I. Rheumatoid cachexia is associated with dyslipidemia and low levels of atheroprotective natural antibodies against phosphorylcholine but not with dietary fat in patients with rheumatoid arthritis:a cross-sectional study. Arthritis Res Ther 2009;11:R37. 19284557 10.1186/ar2643 PMC2688183

[45] Elkan A-C Engvall I-L Cederholm T Hafström I. Rheumatoid cachexia, central obesity and malnutrition in patients with low-active rheumatoid arthritis: feasibility of anthropometry, Mini Nutritional Assessment and body composition techniques. Eur J Nutr 2009;48:315–22. 19333642 10.1007/s00394-009-0017-y

[46] Macaskill P Gatsonis C Deeks J Harbord R Takwoingi Y. Analysing and Presenting Results: Cochrane Handbook for Systematic Reviews of Diagnostic Test Accuracy. Version 1.0. ©The Cochrane Collaboration, 2010.

[47] Santo RC Silva JM Lora PS Moro ALD Freitas EC Bartikoski BJ Cachexia in patients with rheumatoid arthritis: a cohort study. Clin Rheumatol. Epub ahead of print 23 May 2020. DOI:10.1007/s10067-020-05119-y. 32447598

[48] Evans WJ Morley JE Argilés J Bales C Baracos V Guttridge D Cachexia: A new definition. Clin Nutr 2008;27:793–9. 18718696 10.1016/j.clnu.2008.06.013

[49] Muscaritoli M Anker SD Argilés J Aversa Z Bauer JM Biolo G Consensus definition of sarcopenia, cachexia and pre-cachexia:joint document elaborated by Special Interest Groups (SIG) ‘cachexia-anorexia in chronic wasting diseases’ and ‘nutrition in geriatrics’. Clin Nutr 2010;29:154–9. 20060626 10.1016/j.clnu.2009.12.004

[50] Sipilä S Törmäkangas T Sillanpää E Aukee P Kujala UM Kovanen V Muscle and bone mass in middle-aged women: role of menopausal status and physical activity. J Cachexia Sarcopenia Muscle 2020;11:698–709. 32017473 10.1002/jcsm.12547 PMC7296268

[51] Greendale GA Sternfeld B Huang MH Han W Karvonen-Gutierrez C Ruppert K Changes in body composition and weight during the menopause transition. JCI Insight;4. Epub ahead of print 2019. DOI:10.1172/JCI.INSIGHT.124865. PMC6483504 30843880

[52] Ambikairajah A Walsh E Tabatabaei-Jafari H Cherbuin N. Fat mass changes during menopause: a metaanalysis. Am J Obstet Gynecol 2019;221:393–409.e50. 31034807 10.1016/j.ajog.2019.04.023

[53] Baker JF Long J Ibrahim S Leonard MB Katz P. Men Are at Greater Risk of Lean Mass Deficits in Rheumatoid Arthritis. Arthritis Care Res (Hoboken) 2015;67:112. 25048740 10.1002/acr.22396 PMC4280305

[54] Tengstrand B Carlström K Hafström I. Gonadal hormones in men with rheumatoid arthritis--from onset through 2 years. J Rheumatol 2009;36:887–92. 19273455 10.3899/jrheum.080558

[55] Rivier C. Neuroendocrine Effects of Cytokines in the Rat. Rev Neurosci 1993;4:223–38. 9155864 10.1515/revneuro.1993.4.3.223

[56] Rivier C Vale W. In the rat, interleukin-1 alpha acts at the level of the brain and the gonads to interfere with gonadotropin and sex steroid secretion. Endocrinology 1989;124:2105–9. 2651094 10.1210/endo-124-5-2105

[57] Cutolo M Straub RH. Sex steroids and autoimmune rheumatic diseases:state of the art. Nat Rev Rheumatol 2020;16:628–44. 33009519 10.1038/s41584-020-0503-4

[58] Cutolo M Wilder RL. Different roles for androgens and estrogens in the susceptibility to autoimmune rheumatic diseases. Rheum Dis Clin North Am 2000;26:825–39. 11084946 10.1016/s0889-857x(05)70171-x

[59] Schmidt M Kreutz M Löffler G Schölmerich J Straub RH. Conversion of dehydroepiandrosterone to downstream steroid hormones in macrophages. J Endocrinol 2000;164:161–9. 10657851 10.1677/joe.0.1640161

[60] Castagnetta LA Carruba G Granata OM Stefano R Miele M Schmidt M Increased estrogen formation and estrogen to androgen ratio in the synovial fluid of patients with rheumatoid arthritis. J Rheumatol 2003;30:2597–605. 14719200

[61] Fukuda W Omoto A Oku S Tanaka T Tsubouchi Y Kohno M Contribution of rheumatoid arthritis disease activity and disability to rheumatoid cachexia. Mod Rheumatol 2010;20:439–43. 20508962 10.1007/s10165-010-0306-6

[62] Uutela TI Kautiainen HJ Häkkinen AH. Decreasing muscle performance associated with increasing disease activity in patients with rheumatoid arthritis. PLoS One 2018;13:e0194917. 29630626 10.1371/journal.pone.0194917 PMC5890969

[63] Summers GD Deighton CM Rennie MJ Booth AH. Rheumatoid cachexia: a clinical perspective. Rheumatology (Oxford) 2008;47:1124–31. 18448480 10.1093/rheumatology/ken146

[64] Costamagna D Costelli P Sampaolesi M Penna F. Role of Inflammation in Muscle Homeostasis and Myogenesis. Mediators Inflamm; 2015. Epub ahead of print 2015. DOI:10.1155/2015/805172. PMC4609834 26508819

[65] Marcora SM Chester KR Mittal G Lemmey AB Maddison PJ. Randomized phase 2 trial of anti-tumor necrosis factor therapy for cachexia in patients with early rheumatoid arthritis. Am J Clin Nutr 2006;84:1463–72. 17158431 10.1093/ajcn/84.6.1463

[66] Matschke V Murphy P Lemmey AB Maddison P Thom JM. Skeletal muscle properties in rheumatoid arthritis patients. Med Sci Sports Exerc 2010;42:2149–55. 20404765 10.1249/MSS.0b013e3181e304c3

[67] Masuko K. Rheumatoid Cachexia Revisited: A Metabolic Co-Morbidity in Rheumatoid Arthritis. Front Nutr 2014;1:20. 25988122 10.3389/fnut.2014.00020 PMC4428367

[68] Elkan AC Håkansson N Frostegård J Low level of physical activity in women with rheumatoid arthritis is associated with cardiovascular risk factors but not with body fat mass - A cross sectional study. BMC Musculoskelet Disord 2011;12:1–6. 21235741 10.1186/1471-2474-12-13 PMC3027198

[69] Matschke V Murphy P Lemmey AB Maddison PJ Thom JM. Muscle quality, architecture, and activation in cachectic patients with rheumatoid arthritis. J Rheumatol 2010;37:282–4. 20008916 10.3899/jrheum.090584

[70] Giles JT Bartlett SJ Andersen RE Fontaine KR Bathon JM. Association of body composition with disability in rheumatoid arthritis: impact of appendicular fat and lean tissue mass. Arthritis Rheum 2008;59:1407–1415. 18821641 10.1002/art.24109 PMC2670990

[71] Chatzidionysiou K Sfikakis PP. Low rates of remission with methotrexate monotherapy in rheumatoid arthritis:review of randomised controlled trials could point towards a paradigm shift. RMD Open 2019;5:e000993. 31413870 10.1136/rmdopen-2019-000993 PMC6667970

[72] National Guideline Centre. Rheumatoid arthritis in adults:diagnosis and management. London:National Institute for Health and Care Excellence (UK), 2018. 30102507

[73] Ter Borg S de Groot LC Mijnarends DM de Vries JH Verlaan S Meijboom S Differences in Nutrient Intake and Biochemical Nutrient Status Between Sarcopenic and Nonsarcopenic Older Adults-Results From the Maastricht Sarcopenia Study. J Am Med Dir Assoc 2016;17:393–401. 26825685 10.1016/j.jamda.2015.12.015

[74] Kado DM Bucur A Selhub J Rowe JW Seeman T. Homocysteine levels and decline in physical function: MacArthur Studies of Successful Aging. Am J Med 2002;113:537–42. 12459398 10.1016/s0002-9343(02)01269-x

[75] Morgan SL Baggott JE. Folate supplementation during Methotrexate therapy for rheumatoid arthritis. Clin Exp Rheumatol 2010;28:S102–S109. 21044441

[76] Chen C-Y Tsai C-Y Lee P-C Lee S-D. Long-term etanercept therapy favors weight gain and ameliorates cachexia in rheumatoid arthritis patients:roles of gut hormones and leptin. Curr Pharm Des 2013;19:1956–64. 23305268 10.2174/1381612811319100014

[77] Engvall IL Tengstrand B Brismar K Hafström I. Infliximab therapy increases body fat mass in early rheumatoid arthritis independently of changes in disease activity and levels of leptin and adiponectin:a randomised study over 21 months. Arthritis Res Ther 2010;12:R197. 20964833 10.1186/ar3169 PMC2991034

[78] Serelis J Kontogianni MD Katsiougiannis S Bletsa M Tektonidou MG Skopouli FN. Effect of anti-TNF treatment on body composition and serum adiponectin levels of women with rheumatoid arthritis. Clin Rheumatol 2008;27:795–7. 18305977 10.1007/s10067-008-0855-7

[79] Toussirot É Mourot L Dehecq B Wendling D Grandclément É Dumoulin G CBT-506. TNFα blockade for inflammatory rheumatic diseases is associated with a significant gain in android fat mass and has varying effects on adipokines: a 2-year prospective study. Eur J Nutr 2014;53:951–61. 24173963 10.1007/s00394-013-0599-2

[80] Baker JF Cannon GW Ibrahim S Haroldsen C Caplan L Mikuls TR. Predictors of longterm changes in body mass index in rheumatoid arthritis. J Rheumatol 2015;42:920–7. 25834210 10.3899/jrheum.141363 PMC4826749

[81] Hernandez-Baldizon S. [How to effectively use methotrexate in rheumatoid arthritis?]. Reumatol Clin 2012;8:42–5. 22089073 10.1016/j.reuma.2011.01.010

[82] Tian H Cronstein BN. Understanding the mechanisms of action of methotrexate:implications for the treatment of rheumatoid arthritis - PubMed. Bull NYU Hosp Jt Dis 2007;65:168–73. 17922664

[83] Tournadre A Pereira B Dutheil F Giraud C Courteix D Sapin V Changes in body composition and metabolic profile during interleukin 6 inhibition in rheumatoid arthritis. J Cachexia Sarcopenia Muscle 2017;8:639–46. 28316139 10.1002/jcsm.12189 PMC5566648

[84] Toussirot E Marotte H Mulleman D Cormier G Coury F Gaudin P Increased high molecular weight adiponectin and lean mass during tocilizumab treatment in patients with rheumatoid arthritis: a 12-month multicentre study. Arthritis Res Ther 2020;22:224. 32993784 10.1186/s13075-020-02297-7 PMC7523335

[85] Schultz O Oberhauser F Saech J Rubbert-Roth A Hahn M Krone W Effects of inhibition of interleukin-6 signalling on insulin sensitivity and lipoprotein (a) levels in human subjects with rheumatoid diseases. PLoS One;5. Epub ahead of print 2010. DOI:10.1371/JOURNAL.PONE.0014328. PMC3001451 21179199

[86] Hoffman E Rahat MA Feld J Atha J Sieren JC Samei E Effects of tocilizumab, an anti-interleukin-6 receptor antibody, on serum lipid and adipokine levels in patients with rheumatoid arthritis. Int J Mol Sci;20. Epub ahead of print 2 September 2019. DOI:10.3390/ijms20184633. 31540528 PMC6770905

[87] Comee L Taylor CA Nahikian-Nelms M Ganesan LP Krok-Schoen JL. Dietary patterns and nutrient intake of individuals with rheumatoid arthritis and osteoarthritis in the United States. Nutrition 2019;67–68:110533. 10.1016/j.nut.2019.06.014 31445315

[88] Markaki AG Gkiouras K Papakitsos C Grammatikopoulou MG Papatsaraki A Ioannou R Disease Activity, Functional Ability and Nutritional Status in Patients with Rheumatoid Arthritis: An Observational Study in Greece. Mediterr J Rheumatol 2020;31:406. 33521573 10.31138/mjr.31.4.406 PMC7841099

[89] Standley KN Gjertsson I Winkvist A Lindqvist HM. Dietary Habits of Women with Rheumatoid Arthritis Differ from that of Women without the Disease: Results from a Population-Based Study. J Rheum Dis Treat 2019;5:072.

[90] Stone J Doube A Dudson D Wallace J. Inadequate calcium, folic acid, vitamin E, zinc, and selenium intake in rheumatoid arthritis patients: results of a dietary survey. Semin Arthritis Rheum 1997;27:180–5. 9431590 10.1016/s0049-0172(97)80018-2

[91] Kremer JM Bigaouette J. Nutrient intake of patients with rheumatoid arthritis is deficient in pyridoxine, zinc, copper, and magnesium - PubMed. J Rheumatol 1996;23:990–994. 8782128

[92] Gioia C Lucchino B Tarsitano MG Iannuccelli C Di Franco M. Dietary Habits and Nutrition in Rheumatoid Arthritis: Can Diet Influence Disease Development and Clinical Manifestations? Nutrients;12. Epub ahead of print 1 May 2020. DOI:10.3390/NU12051456. 32443535 PMC7284442

[93] Kelepouri D Mavropoulos A Bogdanos DP Sakkas LI. The Role of Flavonoids in Inhibiting Th17 Responses in Inflammatory Arthritis. J Immunol Res;2018. Epub ahead of print 2018. DOI:10.1155/2018/9324357. PMC5859886 29693024

[94] Wilkinson TJ Lemmey AB Jones JG Sheikh F Ahmad YA Chitale S Can Creatine Supplementation Improve Body Composition and Objective Physical Function in Rheumatoid Arthritis Patients? A Randomized Controlled Trial. Arthritis Care Res (Hoboken) 2016;68:729–37. 26414681 10.1002/acr.22747

[95] Marcora S Lemmey A Maddison P. Dietary treatment of rheumatoid cachexia with beta-hydroxy-beta-methylbutyrate, glutamine and arginine:a randomised controlled trial. Clin Nutr 2005;24:442–454. 15896432 10.1016/j.clnu.2005.01.006

[96] Aryaeian N Sedehi SK Khorshidi M Zarezadeh M Hosseini A Shahram F. Effects of hydroalcoholic extract of Berberis Integerrima on the anthropometric indices and metabolic profile in active rheumatoid arthritis patients. Complement Ther Med 2020;50:102331. 32444035 10.1016/j.ctim.2020.102331

[97] Lovell DJ Glass D Ranz J Kramer S Huang B Sierra RI A randomized controlled trial of calcium supplementation to increase bone mineral density in children with juvenile rheumatoid arthritis. Arthritis Rheum 2006;54:2235–42. 16802360 10.1002/art.21956

[98] Jia H Wen Y Aw W Jia Q. Ameliorating Effects of Coriander on Gastrocnemius Muscles Undergoing Precachexia in a Rat Model of Rheumatoid Arthritis: A Proteomics Analysis. Nutr 2021, Vol 13, Page 4041 2021;13:4041. 10.3390/nu13114041 PMC8621435 34836295

[99] Spies CM Straub RH Buttgereit F. Energy metabolism and rheumatic diseases:From cell to organism. Arthritis Res Ther 2012;14:1–10. 22747923 10.1186/ar3885 PMC3446535

[100] Nutsch K Hsieh C. When T cells run out of breath:the HIF-1α story. Cell 2011;146:673–674. 21884928 10.1016/j.cell.2011.08.018

[101] Procaccini C Galgani M De Rosa V Matarese G. Intracellular metabolic pathways control immune tolerance. Trends Immunol 2012;33:1–7. 22075206 10.1016/j.it.2011.09.002

[102] Lacourt TE Vichaya EG Chiu GS Dantzer R Heijnen CJ. The high costs of low-grade inflammation: Persistent fatigue as a consequence of reduced cellular-energy availability and non-adaptive energy expenditure. Front Behav Neurosci 2018;12:78. 29755330 10.3389/fnbeh.2018.00078 PMC5932180

[103] Liu TF Vachharajani VT Yoza BK McCall CE. NAD+-dependent sirtuin 1 and 6 proteins coordinate a switch from glucose to fatty acid oxidation during the acute inflammatory response. J Biol Chem 2012;287:25758–69. 22700961 10.1074/jbc.M112.362343 PMC3406663

[104] Chimenti MS Triggianese P Conigliaro P Candi E Melino G Perricone R. The interplay between inflammation and metabolism in rheumatoid arthritis. Cell Death Dis 2015 69 2015;6:e1887–e1887. 10.1038/cddis.2015.246 PMC4650442 26379192

[105] Reid MB Li YP. Tumor necrosis factor-α and muscle wasting:a cellular perspective. Respir Res 2001;2:269. 11686894 10.1186/rr67 PMC59514

[106] Laviano A Koverech A Mari A. Cachexia: clinical features when inflammation drives malnutrition. Proc Nutr Soc 2015;74:348–354. 25809872 10.1017/S0029665115000117

[107] Binymin K Herrick AL Carlson GL Hopkins S. The effect of disease activity on body composition and resting energy expenditure in patients with rheumatoid arthritis. J Inflamm Res 2011;4:61–66. 22096370 10.2147/JIR.S16508 PMC3218753

[108] Roubenoff R Walsmith J Lundgren N Snydman L Dolnikowski GJ Roberts S. Low physical activity reduces total energy expenditure in women with rheumatoid arthritis:implications for dietary intake recommendations. Am J Clin Nutr 2002;76:774–9. 12324290 10.1093/ajcn/76.4.774

[109] Straub RH. The brain and immune system prompt energy shortage in chronic inflammation and ageing. Nat Rev Rheumatol 2017 1312 2017;13:743–751. 10.1038/nrrheum.2017.172 29021568

[110] Tierney M Fraser A Purtill H Kennedy N. Profile of energy expenditure in people with rheumatoid arthritis. Disabil Health J 2015;8:514–520. 25953350 10.1016/j.dhjo.2015.03.008

[111] Arshad A Rashid R Benjamin K. The effect of disease activity on fat-free mass and resting energy expenditure in patients with rheumatoid arthritis versus noninflammatory arthropathies/soft tissue rheumatism. Mod Rheumatol 2014;17:470–75. 10.1007/s10165-007-0628-1 18084698

[112] Choe JY Park SH Lee H Kwon HH Kim SK. Resting energy expenditure is not associated with disease activity in women with rheumatoid arthritis: cross-sectional study. Korean J Intern Med 2014;29:516. 25045300 10.3904/kjim.2014.29.4.516 PMC4101599

[113] Metsios GS Stavropoulos-Kalinoglou A Panoulas VF Koutedakis Y Nevill AM Douglas KM New resting energy expenditure prediction equations for patients with rheumatoid arthritis. Rheumatology (Oxford) 2008;47:500–6. 18304942 10.1093/rheumatology/ken022

[114] Elkan A-C Engvall I-L Tengstrand B Cederholm T Hafström I. Malnutrition in women with rheumatoid arthritis is not revealed by clinical anthropometrical measurements or nutritional evaluation tools. Eur J Clin Nutr 2008;62:1239–47. 17637600 10.1038/sj.ejcn.1602845

[115] Paget J. Nervous mimicry of organic diseases. Lancet 1873;ii:727–29.

[116] Aletaha D Neogi T Silman AJ Funovits J Felson DT Bingham CO 3rd 2010 Rheumatoid arthritis classification criteria: An American College of Rheumatology/European League Against Rheumatism collaborative initiative. ARTHRITIS Rheum 2010;62:2569–81. 20872595 10.1002/art.27584

[117] Piccoli A Nescolarde LD Rosell J. Análisis convencional y vectorial de bioimpedancia en la práctica clínica. NEFROLOGÍA 2002;XXII:228–38. 12123122

[118] Boers M Verhoeven AC Markusse HM Westhovens R van Denderen JC van Zeben D Randomised comparison of combined step-down prednisolone, methotrexate and sulphasalazine with sulphasalazine alone in early rheumatoid arthritis. Lancet (London, England) 1997;350:309–18. 9251634 10.1016/S0140-6736(97)01300-7

